# Longer telomeres in chronic, moderate, unconjugated hyperbilirubinaemia: insights from a human study on Gilbert’s Syndrome

**DOI:** 10.1038/srep22300

**Published:** 2016-03-01

**Authors:** Anela Tosevska, Christine Moelzer, Marlies Wallner, Milan Janosec, Ursula Schwarz, Carina Kern, Rodrig Marculescu, Daniel Doberer, Wolfram Weckwerth, Karl-Heinz Wagner

**Affiliations:** 1 Research Platform Active Ageing, University of Vienna, Althanstrasse 14, 1090, Vienna, Austria; 2 Department of Nutritional Sciences, University of Vienna, Althanstrasse 14, 1090 Vienna, Austria; 3 Institute of Dietetics and Nutrition, University of Applied Sciences, FH JOANNEUM, Alte Poststraße 149, 8020 Graz, Austria; 4 Institute of Pharmacology, Medical University of Vienna, Waehringer Str. 13a, 1090 Vienna, Austria; 5 Clinical Institute of Laboratory Medicine, Medical University of Vienna, Waehringer Guertel 18–20, 1090 Vienna, Austria; 6 Department of Clinical Pharmacology, Medical University of Vienna, Waehringer Guertel 18–20, 1090 Vienna, Austria; 7 Department of Ecogenomics and Systems Biology, University of Vienna, Althanstrasse 14, 1090 Vienna, Austria; 8 Vienna Metabolomics Center (VIME), University of Vienna, Althanstrasse 14, 1090, Vienna, Austria

## Abstract

Bilirubin (BR) is a natural endogenous compound with a potent bioactivity. Gilbert’s Syndrome (GS) is a benign hereditary condition of increased unconjugated bilirubin (UCB) in serum and serves as a convenient model for studying the effects of BR in humans. In absence of liver disease, increased UCB levels are inversely associated to all-cause mortality risk, especially from cardiovascular diseases (CVDs). On the other hand, telomere malfunction is linked to a higher risk of CVDs. To our knowledge, there is no data on whether UCB is linked to telomere length in healthy or diseased individuals In the present study we have observed a relationship between mildly increased serum UCB and telomere length. We used an *in vivo* approach, assessing telomere length in PBMCs from individuals with GS (n = 60) and matched healthy controls (n = 60). An occurrence of longer telomeres was observed in male individuals chronically exposed to increased UCB, as well as in Gunn rats, an animal model of unconjugated hyperbilirubinaemia. Previously identified differences in immunomodulation and redox parameters in individuals with GS, such as IL-6, IL-1β and ferric reducing ability of plasma, were confirmed and proposed as possible contributors to the occurrence of longer telomeres in GS.

Gilbert’s Syndrome is a condition where mild unconjugated hyperbilirubinaemia is the main phenotypical characteristic^1^. Bilirubin is a natural and ubiquitous bile pigment that has caused a lot of contradictions throughout the years because of its double-faced nature^2^,^3^. It is the end-product of haem catabolism in mammals, formed in subsequent enzymatic reactions in which haem is transformed into biliverdin (BV) and then to bilirubin^4^,^5^. A graphical representation of the processes has been given by Wagner *et al.*^6^.

From an evolutionary perspective, it is puzzling why the BV-BR system has developed, since BR is a potent neurotoxin if present at very high concentrations in the circulatory system^7^. Biliverdin is hydrophilic and can be easily excreted^5^. In fact, BV is the end product of heme catabolism in most birds, reptiles and amphibians^8^. Unconjugated BR, on the other hand, is highly hydrophobic, making its excretion impossible without prior conjugation, a process that yields hydrophilic conjugated BR. This process in mammals is mediated by the UGT1A1 isoenzyme.

Gilbert’s Syndrome is associated with genetic variations in the UGT1A1 gene promoter region, of which the so-called UGT1A1*28 is the most common among Caucasians. This polymorphism is characterized by insertion of an additional TA repeat in the TATA box of the promoter, yielding 7 TA repeats instead of the common 6, and leading to a decreased expression and activity of the UGT enzyme^9^. Since the isoenzyme A1 exhibits the highest affinity towards endogenous BR^10^, its conjugation becomes hindered resulting in increased concentrations of the hydrophobic unconjugated BR (UCB) in the serum of affected individuals, leading to increased measurable UCB and total BR concentrations.

Epidemiological studies indicate a strong association between increased serum BR and decreased incidence of age-related metabolic and cardiovascular diseases^11^,^12^. In addition, individuals with GS have been found to exhibit delayed all-cause mortality, especially that from CVDs^11^,^13^ (summarized in^6^,^14^). In an earlier study, we have shown that GS individuals exhibit a serum lipid profile indicative of lower CVD risk as compared to age-matched controls^15^. These studies investigate mainly the unconjugated portion of serum bilirubin and total serum bilirubin. However, the underlying mechanism of cardiovascular protection remains unclear.

Bilirubin has been described as a potent antioxidant with immune-modulatory activity^16^. This could in turn explain the previous epidemiological findings, as aging and chronic disease are associated with increased baseline inflammation and redox-imbalance^17^,^18^. Another common hallmark of aging is increased telomere attrition^19^. Telomeres are nucleo-protein structures capping the ends of eukaryotic chromosomes. Due to the end-replication problem occurring during mitosis, telomeres cannot be replicated entirely and shorten after each cell division, which is exacerbated by increased oxidative stress^20^,^21^,^22^. A link between accelerated telomere shortening and a number of age-associated diseases and conditions has already been established^23^,^24^. Bilirubin might play a role in maintaining a balanced redox environment delaying telomere attrition and disease onset.

Here we present a case-control study of healthy age- and gender-matched GS individuals and controls within a broadly defined age range. The aim was to provide a link between moderately increased unconjugated bilirubin and telomere length *in vivo* and to propose an involvement of telomeres as mediators of bilirubin signalling in healthy aging.

## Results

### Characteristics of the study population

A total of 120 participants were allocated to two groups: case and control, according to the levels of UCB as determined by high-performance liquid chromatography (HPLC), with a cut-off point at 17.1 μmol/L. Each case was matched with an age- and gender-appropriate control. The study characteristics are depicted in Table 1.

### Telomere length analysis

**Figure 1:**
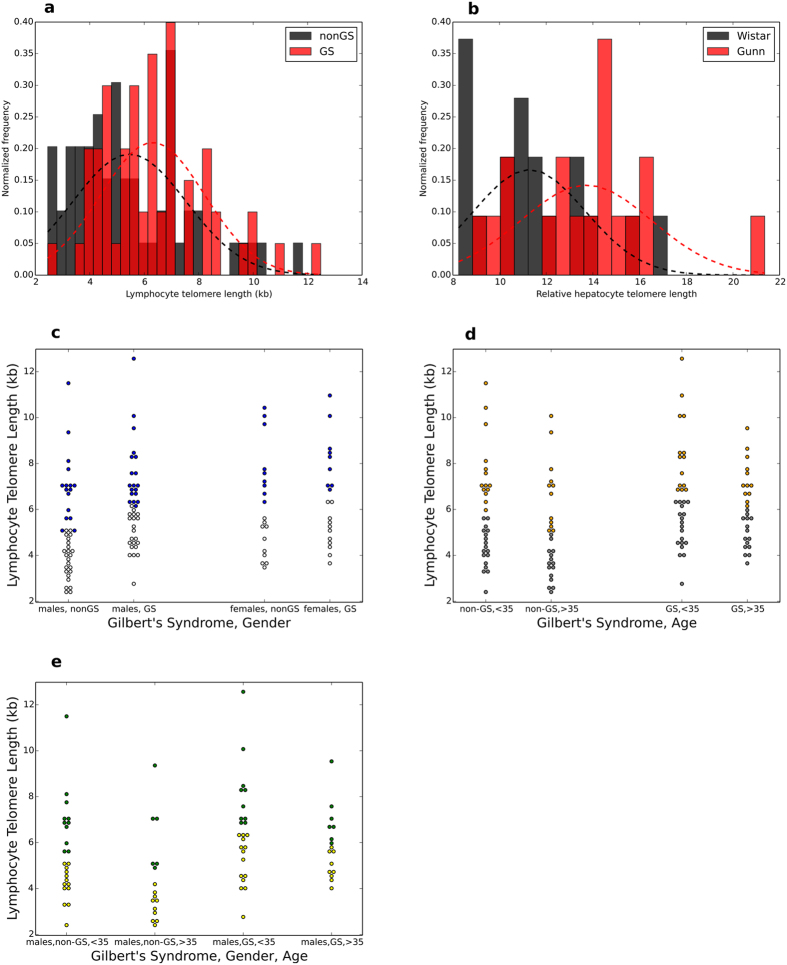
Telomere length distribution in human and animal samples. (**a,b**) Dark red bars represent frequency overlap between the groups. (**c,d,e**) Different colours represent values below and above the group mean (**a**) Distribution of absolute lymphocyte telomere length in Gilbert’s individuals; P = 0.01, Mann Whitney U test; P = 0.031, independent samples t-test, two-sided. (**b**) Distribution of relative liver telomere length in homozygous Gunn rats vs. heterozygous Wistar rats, P = 0.013, Mann Whitney U test; P = 0.01, independent samples t-test, two-sided. (**c**) Lymphocyte telomere length in GS and non-GS, age groups; P = 0.024, Kruskall-Wallis 1-way ANOVA; P = 0.053 for ≥35 years old GS vs. non-GS, independent samples t-test, two-sided. (**d**) Lymphocyte telomere length in GS and non-GS, gender; P = 0.013, Kruskall-Wallis 1-way ANOVA; P = 0.013 for GS males vs. non-GS males. (**e**) Lymphocyte telomere length in GS and non-GS, age groups, males only; P = 0.01, Kruskall-Wallis 1-way ANOVA; P = 0.02 for ≥35 years old GS vs. non-GS males, independent samples t-test, two-sided.

Mean telomere length (TL) was greater in GS subjects (6.25 kb ± 1.97, mean ± s.d.) compared to controls (5.47 kb ± 2.09, P = 0.033; paired t-test), which became more pronounced after correcting for age and gender (P = 0.002 for the whole model, P = 0.02 for GS phenotype). The differences in TL distribution are shown in Fig. 1a. We further examined liver tissue of Gunn rats, an animal model of hyperbilirubinemia^25^, vs. healthy Wistar rats. Gunn rats exhibited significantly longer telomeres (13.71 ± 2.88 vs. 11.28 ± 2.46; P = 0.01; relative units; Fig. 1b).

We then set to assess which variables most markedly affect TL, independent of the GS condition. Age and gender cumulatively contributed to an almost negligible variance in telomere length data (6.7%, P < 0.05). As expected, and consistent with previous findings^26^, TL decreased with age at a rate of around 30 bases per year, after correction for gender. On the other hand, females tended to have longer telomeres than males, with a difference of around 1 kb after age correction (P = 0.025). When dividing the cohort according to gender, a significant difference between GS and controls was only evident in males (P = 0.01, Fig. 1c,e, supplementary Table S1). No gender-specific difference concerning TL was observed in rats (Supplementary Fig. S1b,c, supplementary Table S2).

### Unconjugated bilirubin and telomere length

As expected, UCB was significantly different between the groups (Table 1). There was no effect of gender on UCB in the entire population, however, males had slightly higher levels in the GS group (supplementary Table S1). UCB levels had a tendency to decrease with age in the control group (r = −0.274, P = 0.034, supplementary Table S3).

**Figure 2:**
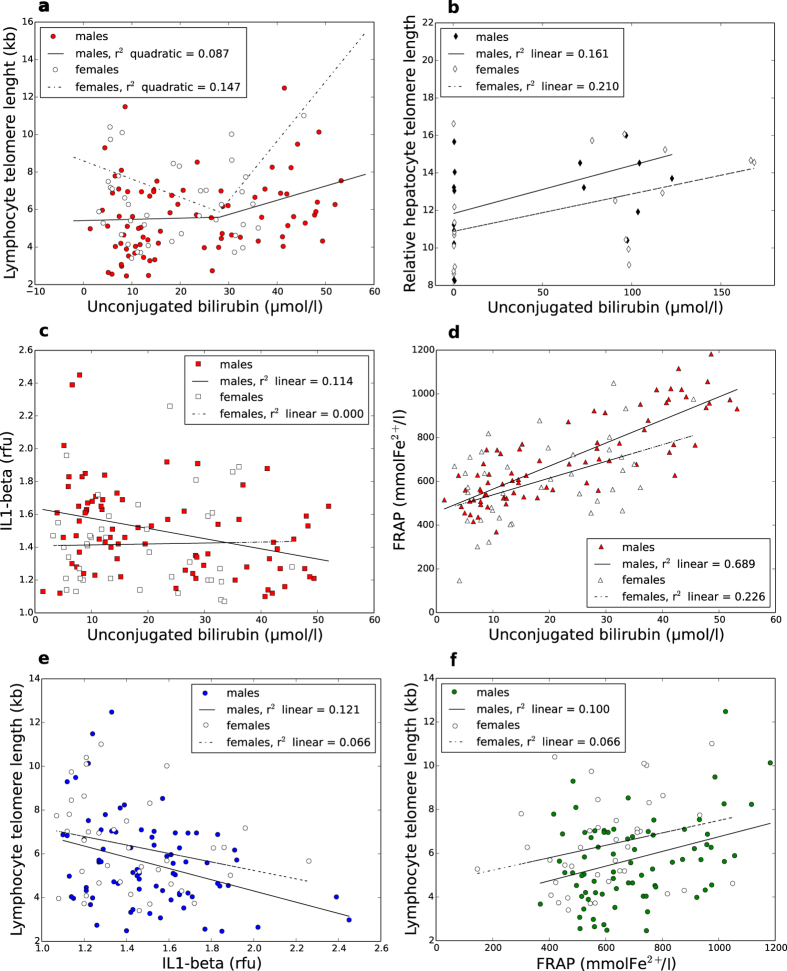
Correlation of serum UCB with telomere length, serum antioxidant capacity and intracellular interleukins in humans; plasma UCB and hepatocyte telomere length in rats. (**a**) Association between UCB and telomere length in males and females; quadratic model: P(males) = 0.03, P(females) = 0.053; linear model: P(males) = 0.012, P(females) = 0.713. (**b**) Plasma UCB and relative hepatocyte telomere length in rats; P(males) = 0.110, P(females) = 0.048. (**c**) UCB and IL1β; P(males) = 0.003, P(females) = 0.879. (**d**) UCB and FRAP; P(males) < 0.001, P(females) = 0.002. (**e**) IL1β and lymphocyte telomere length; P(males) = 0.002, P(females) = 0.110. (**f**) FRAP and lymphocyte telomere length; P(males) = 0.005, P(females) = 0.111.

Serum UCB levels strongly correlated with the ferric reducing ability of plasma (FRAP) in both males and females (r = 0.729, P < 0.001 Fig. 2d), but not with other markers of oxidative stress such as GSH/GSSG or malondialdehyde. Baseline inflammation markers in serum such as CRP and SAA were only weakly inversely associated with UCB (r = −0.2, P = 0.02 and r = −0.19, P = 0.04, respectively), whereas IL-6 and IL-1β in monocytes showed a stronger inverse correlation (r = −0.301, P = 0.001 and r = −0.211, P = 0.023 respectively) which remained significant after age correction. After gender stratification, a strong inverse relationship with IL-1β was evident in males, but not in females (Fig. 2c).

Intracellular IL-1β was by far the best single predictor of telomere length (Fig. 2a,d,e). When combined with the variables age, gender and FRAP in total a contribution of 22% to TL variability in the entire study population became evident in the model (P < 0.001). UCB and TL were only weakly correlated after correcting for age and gender, though a U-shaped model could fit the data more precisely (Fig. 2a). Other parameters affected by the GS condition such as plasma iron, BMI or blood pressure showed no correlation with telomere length.

### UGT1A1 genotype and telomere length

**Figure 3:**
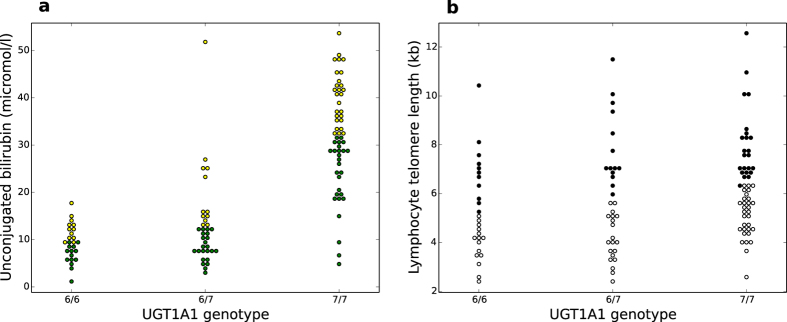
UGT1A1 genotypes and their influence on telomere length. (**a**) UCB concentration distribution according to genotype, P < 0.001, Kruskall-Wallis 1-way ANOVA. (**b**) Telomere length according to genotype P = 0.034, Kruskall-Wallis 1-way ANOVA.

To better characterize the nature of the hyperbilirubinaemia, we performed UGT1A1 genotyping of the human subjects. While the GS group almost entirely consisted of individuals with the 7/7 genotype (homozygous for the allele with 7 TA-repeats, sup. Table S4) more than 50% of the control subjects turned out to be heterozygous carriers of the UGT1A1 7 allele, which often presents with intermediately high UCB levels (Fig. 3b). UCB concentrations differed significantly when comparing the 7/7 genotype to the 6/6 and 6/7 UGT1A1 genotype (P < 0.001, sup. Table S5). No significant difference was present between the 6/6 and 6/7 genotype before or after adjustment for multiple comparison. The groups did not differ significantly in age and gender distribution (sup. Table S5).

Telomere length across genotypes showed a similar pattern as UCB, with heterozygous carriers showing an intermediate mean telomere length. The difference remained significant only between 6/6 and 7/7 homozygous subjects (P = 0.044, after adjustment for multiple comparison, Fig. 3c, supplementary Table S5).

### PCA, multivariate correlations and clustering

**Figure 4:**
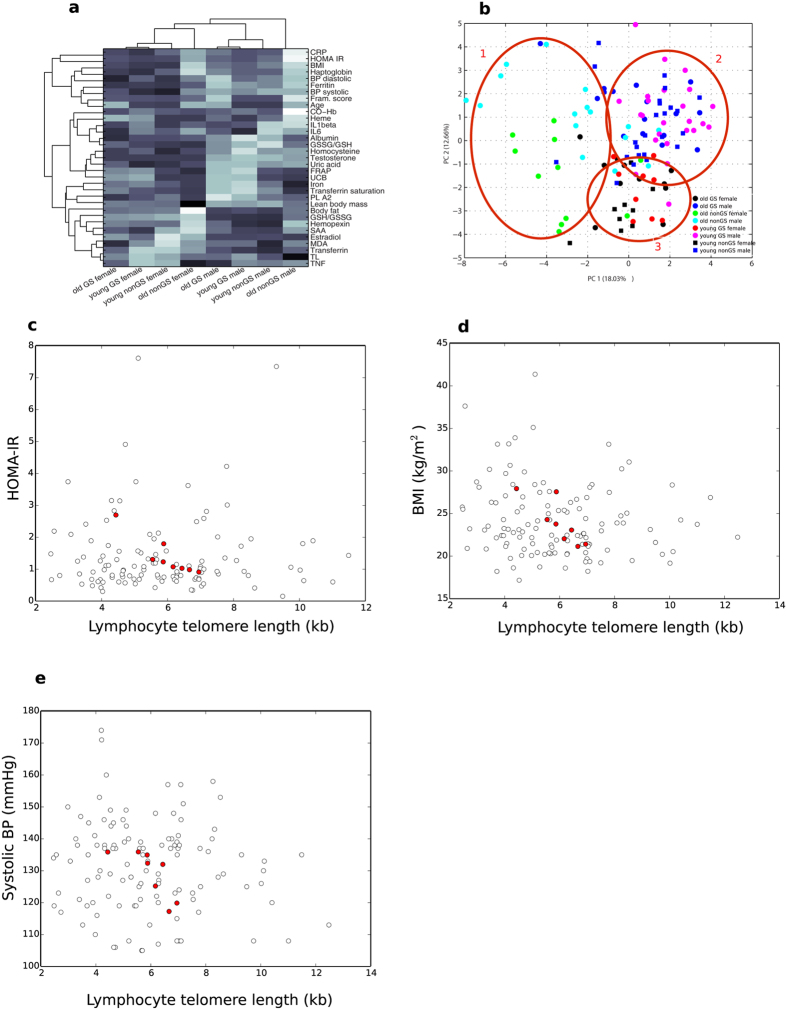
COVAIN analyses revealed distinct clustering among the eight study groups, divided according to age, gender and GS phenotype. Males and females form two distinct clusters with a small distance between “young” (<35 years old) individuals and “old” (≥35 years old) GS individuals, whereas there is a clearly greater distance of “old” control subjects. (**a**) Heat map shows clustering of groups and contributing variables. For individual differences in variables between groups refer to supplementary Tables S1 and S2. (**b**) Principal components analysis contribute to formation of three distinct clusters (marked with red circles). Circle 1: old, non-GS males and females; circle 2: males, young GS and non-GS and old GS; circle 3: females, young GS and non-GS and old GS. (**c**) Pearson correlation between TL and HOMA-IR, white circles represent all data, P > 0.1; red circles represent group mean, r = −0.906, P = 0.002. (**d**) Pearson correlation between TL and systolic blood pressure, white circles represent all data, P > 0.1; red circles represent group mean, r = −0.762, P = 0.028. (**e**) Pearson correlation between TL and BMI, white circles represent all data, P > 0.1; red circles represent group mean, r = −0.827, P = 0.011.

We further performed a more in-depth multivariate principal component analysis (PCA) and bi-clustering using COVAIN^27^. Bi-clustering of the data revealed a clear discrimination between GS and non-GS individuals (Fig. 4a,b). The main separation is seen as two clusters of female and male subjects. Young individuals (≤35 years of age, GS and non-GS) and older GS subjects (≥35 years) always grouped closely together, whereas the older controls deviated more in their characteristics (Fig. 4a,b). This was as well evident for telomere length and observed previously for lipid metabolism^15^. Additionally, analysis revealed a strong negative correlation between TL and blood pressure, BMI and HOMA-IR as outcome variables, when considering the mean group values (Fig. 4c–e). This was not evident when employing a simple bivariate correlation.

## Discussion

Our findings reveal a novel characteristic of Gilbert’s Syndrome, potentially related to a chronic exposure to moderately increased unconjugated serum bilirubin. We show that individuals with a GS phenotype have on average longer telomeres compared to age- and gender-matched controls. This difference appears to be more pronounced with age, suggesting a reduced or slower telomere attrition in GS.

The liver is the key organ involved in heme catabolism that is affected by differences in UGT1A1 expression and UCB accumulation. However, due to ethical guidelines and based on the invasiveness of liver biopsies, human hepatocytes were inaccessible in this study. Using rat liver tissue instead, we could observe that telomere length distribution in GS individuals was clearly reflected by the animal model. Interestingly, in rats the differences were evident, despite the very young age at sacrifice (7–8 weeks). However, at this age, telomerase is still active in rat hepatocytes^28^, indicating there could be a different telomere maintenance mechanism compared to humans.

Due to a lack of human liver samples and rodent PBMCs, respectively, we can only presume that TL is retained across different tissues, as is supported by the findings of Daniali *et al.*^26^.

The difference in telomere length between GS and non-GS individuals was only evident in males. This goes in line with previous findings on the dimorphic effect of increased serum BR as a protective agent against CVDs^6^. Steroid hormones, such as 17β-estradiol are also glucuronidated by the UGT1A1 isoenzyme^10^, but with a lower affinity compared to BR. Hence, we expected to observe an increase in serum concentrations of 17β-estradiol in GS. In addition, oestrogen is a known inducer of telomerase, the main enzyme involved in telomere maintenance. However, serum 17β-estradiol concentrations could not explain the occurrence of longer telomeres in GS males (sup. Table S1). Thus, the reasons for the observed gender differences remain unclear. In the rodent models, increased telomere length accompanied hyperbilirubinaemia in both male and female animals. As mentioned above, comparison to the human GS condition is difficult, as telomerase is highly active in rat liver.

The most plausible hypothesis explaining slower telomere shortening in GS, is that UCB via its immune-modulatory activity leads to slower cell turnover and hence, a slower exhaustion of hematopoietic stem cells^29^. This, combined with the antioxidant potential of BR could explain most of the differences observed in TL. A strong direct relationship between serum UCB and TL could not be established, likely due to the transient nature of measurable UCB concentrations, which are subject to day-to-day fluctuations. On the other hand, changes in TL are steady and can be only observed on a long-term basis. UCB might affect the immune response by downregulating intracellular production of cytokines, as evident from the lower levels of IL6 and IL-1β in monocytes from GS individuals, in particularly males (sup. Table S1). A telomere shortening effect of IL-1β has already been observed as a result of faster cell turnover influenced by increased baseline inflammation^30^.

Although it is tempting to propose a strong intracellular immune-modulatory effect of UCB, there is no data on uptake of bilirubin by immune cells *in vivo*. Although UCB can be readily taken up by PBMCs in Gunn rats (Fig. 3, supp. Fig. S2), we could not detect it in PBMCs from GS individuals. This might be in turn due to the low levels of free BR (Table 1), which are able to enter the cells and are much lower than the limit of detection (18 nmol/l) of the HPLC method used^31^. In addition, even in Gunn rats, lymphocytes showed significantly lower affinity to UCB uptake compared to hepatocytes and colonocytes (supplementary Fig. S2b).

While increased UCB levels are so far the only identified outcome in Gilbert’s Syndrome, we cannot exclude that UGT1A1 mutations might exhibit other effects on human health. UGT1A1 genotyping confirmed previous findings of high occurrence of the UGT1A1*28 allele even in the control population not exhibiting GS phenotype, as more than 50% of the controls have proven to be heterozygous carriers of the allele^32^,^33^. Additionally, a small number of heterozygous individuals showed a GS phenotype (sup. Table S4). However, taken in account the general characteristics such as UCB, BMI and Iron (sup. Table S5), the heterozygous carriers appeared to be phenotypically closer to the 6/6 than to the 7/7 individuals, indicating that both copies of the aberrant UGT1A1 promoter are necessary for beneficial health outcomes related to GS.

The present study aimed to investigate differences between healthy individuals with no history or present indication of a CVD. Even though we could identify a mildly increased risk for cardiovascular events (Framingham 10-year risk score, Table 1) in the control population compared to the GS population, we could not attribute the observed difference to either UCB or telomere length differences.

An interesting parallel between the importance of telomere and bilirubin biology in CVD protection can be made looking at previous reports^34^,^35^,^36^. These studies employ a method of exogenously introduced telomerase/bilirubin treatment on myocardial ischemia/infarction injury, resulting in a reduced cardiac muscle damage, possibly by modulating cell death. Bilirubin might offer cardiac protection via telomere targeting and more studies in this direction should be performed to test this possibility.

Being the first study reporting this results and dealing with a relatively small sample size, additional similar studies are needed in order to confirm our findings. Besides, the current study design is characterized by a heterogeneous age range, and a larger sample size would compensate for its effects. In order to properly assess telomere dynamics in GS, a longitudinal approach following the same individuals over a prolonged time period, could be employed. It is of major importance to take a more in-depth look at the mechanisms behind the telomere length differences, and while our proposed model of reduced inflammation and cell turnover is very plausible, specific pathways remain to be uncovered. In addition, in order to properly assess the implications of the UCB-telomere length axis in CV health, a study investigating telomere length and Gilbert’s Syndrome in CVD patients should be conducted.

In conclusion, we could observe a further link between Gilbert’s Syndrome, and possibly unconjugated bilirubin, and longevity, with emphasis on healthy aging. This work is important when considering bilirubin as a natural therapeutic in fighting cardio-metabolic diseases^14^, as it emphasizes a long-term benefit on a cellular and molecular level.

## Materials and Methods

### Subject recruitment and sample preparation

This study was a case-control study (60 patients in each group) at a single centre in Austria. The study was performed at the Department of Clinical Pharmacology of the Medical University of Vienna and subjects (both control and GS) were recruited between 06/2014 and 01/2015 by direct advertising (bulletin boards, posters and flyers) and from the department’s subject database.

A total of 128 subjects (men and women) from the general population were recruited after a screening examination including liver enzyme values for γ-glutamyl transferase (γ-GT), alanine transferase (ALT), aspartate aminotransferase (AST), lactate dehydrogenase (LDH), alkaline phosphatase, and blood count including reticulocytes, haemoglobin, and haematocrit. Inclusion criteria were normal liver function, absence of acute and chronic disease, and age between 20 and 80 years. Subjects with liver, heart or kidney conditions, haemolysis, diabetes, cholelithiasis, organ transplants, history of cardiovascular disease (CVD), cancer, smoking, excessive physical activity, and any medication that might alter liver metabolism as well as vitamin supplementation (4–5 weeks prior the blood sampling) were excluded. As an important diagnostic procedure, subjects were required to complete 400 kcal restricted fasting-protocol on the day preceding blood sampling, leading to increased serum UCB levels in absence of liver disease^37^. The criterion for group allocation (Gilbert’s Syndrome or control group), was based on a fasting serum unconjugated bilirubin (UCB) concentration of ≥ or <17.1 μmol/L respectively, measured by high-performance liquid chromatography (HPLC). Finally, subjects were stratified for the GS and control group and matched by age and gender to result in 40 men and 20 women in both groups, respectively, which reflects the occurrence of GS phenotype in the population^15^.

The study was approved by the Ethical Committee of the Medical University of Vienna and the General Hospital of Vienna (#1164/2014) and conducted in accordance with the approved guidelines by the Declaration of Helsinki. All subjects provided written informed consent.

For each subject, fasting blood samples were collected on a single occasion, a maximum of two week following the screening test. Samples were drawn by venepuncture and a peripheral venous catheter, into EDTA, Li-Heparin and serum vacutainers (Vacuette K_2_EDTA and Z Serum Sep respectively, Greiner Bio-one GmbH). Samples were stored in cool and dark and subsequently processed, to yield aliquoted fractions thereof (whole blood, plasma, serum). The aliquots were stored at −80 °C until further analysis.

Peripheral blood mononuclear cells (PBMCs) were isolated from EDTA blood using Leucosep separation tubes (Greiner bio one), by centrifugation according to the manufacturer’s instructions and washed twice with ice-cold PBS. Cell count and viability was estimated using the Trypan blue exclusion test on a Countess Automated cell counter (Life Technologies). Cells were aliquoted in freezing medium (FBS + 10% DMSO) and cooled gradually to −80 °C using CoolCell (Biozym).

### Anthropometric measurements

Standing height was measured without shoes to the nearest 0.5 cm with a commercial stadiometer (Seca, Hamburg, Germany), and body mass was evaluated with a digital scale (BWB 700, Tanita, Amsterdam, Netherlands) to the nearest 0.1 kg with subjects lightly dressed and barefoot. BMI was calculated as the ratio between the weight measured in kilograms and the square of the height measured in meters. To determine body composition (muscle and fat mass) Bioelectric Impedance Analysis (BIA) was used, which has been shown to provide reliable data of body composition in comparison to Dual-Energy X-ray Absorptiometry (DXA)^38^. BIA was performed in the morning after an overnight fast using a BIA Analyzer 2000-S (Data-Input GmbH, Darmstadt, Germany).

### Blood biochemistry

Liver enzymes (AST, ALT, γ-GT, LDH), iron, ferritin, transferrin, hemopexin, haptoglobin, homocysteine, hormones (estradiol, testosterone, TSH, triiodthyronine, thyroxin) were analysed in the routine core laboratory of the hospital (Olympus 5400 clinical chemistry analysers from Beckman Coulter) and measured on the day of blood sampling. Carboxy haemoglobin (CO-Hb) was measured directly from heparinized syringes, using a blood gas analyser ABL 700 (Radiometer, Brønshøj, Denmark).

### Unconjugated bilirubin measurement by HPLC

Serum samples for UCB measurement were stored in dark tubes, immediately after separation, as described previously^37^. Unconjugated bilirubin and heme were measured in serum samples, using a high-performance liquid chromatograph (Merck, Hitachi, LaChrom, Vienna, Austria) equipped with a photodiode array detector (PDA, Shimadzu,) and a Fortis C18 HPLC column (4·6 × 150 mm, 3 μm) with a phenomenex C18 HPLC guard column (4·0 × 3·0 mm). Sample preparation and analysis were performed as previously published^37^. Unconjugated bilirubin and haemin (both Frontier Scientific Europe, Carnforth, Lancashire, UK) served as external standards.

Free bilirubin was calculated from serum UCB and albumin levels, using a formula kindly provided by Dr. Silvia Gazzin and Dr. Claudio Tiribelli.

### Gunn rat liver sample preparation

Hyperbilirubinaemic Gunn rats (20 in total, 9 males and 11 females), homozygous for a mutation in *UGT1A1*, and the same number of normobilirubinaemic Wistar rats, heterozygous for a mutation in *UGT1A1*, were obtained from Charles University in Prague (Prague, Czech Republic) and acclimatized in the breeding facility of the Medical University of Vienna (Himberg, Austria). The animals were housed in plastic cages (Macrolon type IV; Techniplast), under standard conditions (24 ± 1 °C, humidity 50 ± 5 °C, 12 h light/dark cycle) and fed with a standard diet (ssniff R/M-H Extrudat; ssniff Spezialdiäten) and *ad libitum* access to fresh water. The animals were sacrificed at the 7–8-week of age. The study was approved by the committee of animal experiments of the Austrian Federal Ministry of Science and Research (BMF-66.006/0008-II/3b/2011), and was carried out in accordance with the approved guidelines. Frozen liver samples, were used for DNA extraction. After thawing, not more than 20 mg rat liver was harvested and homogenized in ALT buffer (DNeasy Blood and Tissue Kit, QIAGEN) and Proteinase K. The samples were incubated at 37 °C overnight, until complete lysis. DNA extraction was completed the next day, using the kit manufacturer’s standard protocol. Purity of the samples was determined by Nanodrop 2000c spectrophotometer (Thermo Scientific), and double stranded DNA concentration was measured by QuantiFluor® dsDNA System (Promega).

For UCB measurement, cells and tissue samples were homogenized in HPLC mobile phase and prepared as described in the previous paragraph. Protein concentrations were measured using the Bradford reagent (Sigma Aldrich), as instructed by the manufacturer.

### DNA extraction from PBMCs and concentration measurement

DNA from human PBMCs was extracted from two million cells, using DNeasy Blood and Tissue Kit (QIAGEN), according to manufacturer’s instructions. DNA concentration was measured using the QuantiFluor® dsDNA System (Promega), as a plate-based assay. A random subset of samples were measured using NanoDrop 2000c spectrophotometer (Thermo Scientific) and DNA integrity was estimated by agarose gel electrophoresis. Samples were stored at −20 °C, for no longer than 6 months prior to analysis.

### Absolute telomere length measurement in PBMCs by qPCR

Telomere length measurement was performed as described^39^, with modifications. Shortly, SYBR Select Master Mix (Life Technologies) was used to amplify telomeric sequences and SCG (36b4). Primers were used at a final concentration of 100 nM. Primer sequences are shown in supplementary Table S6. Genomic DNA samples were diluted to a concentration of 2.5 ng/μl, and 4 μl were used in each reaction (10 ng/reaction). 84-base oligonucleotide standards were diluted to a stock solution of 50 pg/μl. To generate a telomere standard curve, 10-fold serial dilutions of the stock solution were prepared. For the SCG standard curve, the stock solution was diluted to 0.5 ng/μl, and serial dilutions were prepared thereof. All samples and standards were run in triplicate. Assays were run on an ABI 7300 Real-Time PCR System (Life Technologies), using a transparent 96-well plate and optical seals (4titude®).

Analyses were performed at a manual threshold of 2.461 for both targets, with a qPCR efficiency ranging between 90–110%. Samples with a standard deviation exceeding 0.5 Ct were excluded from the analysis. Absolute telomere length was calculated as kb/telomere as previously described^39^.

### Relative telomere length measurement in liver by qPCR

For measuring rat liver TL, we used a similar method to the one described above, on a QuantStudio™ 6 Flex Real-Time PCR System (Thermo Fisher), using a 384-well block, in a single run. Relative telomere length was calculated as 2^dCt (dCt = Ct(Telomere) – Ct(36b4)).

### Intracellular cytokines

IL-6, IL-1b and TNF were measured in monocytes following the standard intracellular protein staining protocol (BD) using a FACSCalibur (BD) flow cytometer. Briefly, heparinized whole blood was treated with brefeldin A solution for 4 h, to block cytokine excretion. Red blood cells were lysed (Cell Lysis Solution 1:10, BD) and cells were stained with CD14 surface antibody (Biolegend). In the next step, cells were permeabilised (Permeabilizing Solution 2, 1:10, BD), and treated with intracellular antibodies against IL-6, IL-1b and TNF (BD). Cells were measured immediately following fixation and relative fluorescence intensity was estimated against isotype control.

### Ferric Reduction Ability of Plasma (FRAP) assay

The reducing capacity in serum was measured using the FRAP assay^40^. The method is based on the reduction of ferric (Fe^3+^-) tripyridyl triazine resulting in the blue-coloured ferrous (Fe^2+^-) tripyridyl triazine complex. All samples were analysed at wavelength of 593 nm, on a BMG FLUOstar OPTIMA Microplate Reader (BMG LABTECH GmbH) in triplicate in a 96-well format. Ascorbic acid served as standard, and results were expressed as Trolox equivalents in μmol/l.

### GSH/GSSG

Oxidized and reduced glutathione were measured using N-Ethylmaleimide and O-phthalaldehyde, as described previously^41^. All samples were analysed in triplicates using external GSSG and GSH standards, in a 96-well plate fluorometer (BMG FLUOstar OPTIMA Microplate Reader, BMG LABTECH GmbH).

### MDA

MDA levels were determined in plasma as described earlier^42^. The samples were neutralized after heating (60 min, 100 °C) with methanol/NaOH, centrifuged (3 min, 3000 rpm) and MDA was measured with high-performance liquid chromatography (HPLC) (excitation: λ 532 nm, emission: λ 563 nm, LaChrom Merck Hitachi Chromatography System, Vienna, Austria; HPLC column (125 × 4 mm, 5 μm; Merck, Vienna, Austria). Each sample was analysed in duplicate.

### UGT1A1 Genotyping

DNA for UGT1A1 genotyping was extracted from whole blood, using the QIAsymphony SP automated system with QIAsymphony DSP DNA Midi Kit (QIAGEN), according to manufacturer’s instructions.

Analyses were performed as described previously^43^. Primers and probes were used as 10 μM working solutions. LightCycler FastStart DNA Master HybProbe Mix (Roche) was used on a LightCycler 480 Instrument II (Roche). Alleles were determined according to the melting curves obtained.

### Statistical analysis

The current study is of explorative nature without a specified primary outcome parameter. The sample size calculation was done taking in account data from a previous study in hyperbillirubinemic humans^44^ where based on the assumption of a difference in DNA damage of 12 ± 2.5% vs 10 ± 2.5% (non-GS vs GS) with a type I error of 0.05 and type 2 error of 0.1 a sample size of 33 persons per group was calculated. For the current study we doubled the sample size.

Statistical analyses were done using SPSS (version 21, IBM). Paired Student’s t-test or Mann Whitney U test were performed to evaluate the differences between cases and controls. Bivariate correlations were evaluated using either Pearson or Spearman coefficients, depending on data distribution. PCA and bi-clustering analysis were performed using COVAIN toolbox for MATLAB^27^. The bi-clustering uses average linkage of Euclidean distance between groups as the metric. Plots were created using the Matplotlib library for Python 2.7 and 3.3.

## Additional Information

**How to cite this article**: Tosevska, A. *et al.* Longer telomeres in chronic, moderate, unconjugated hyperbilirubinaemia: insights from a human study on Gilbert’s Syndrome. *Sci. Rep.* **6**, 22300; doi: 10.1038/srep22300 (2016).

## Supplementary Material

Supplementary Dataset 1

Supplementary Information

## Figures and Tables

**Table 1:** Characteristics of the study population (demographics, variables defining haem and iron metabolism, anthropometrical measurements and CVD risk parameters).

	Gilbert’s Syndrome (n = 49–60)	Controls (n = 49–60)
Age (years)	37 ± 14	37 ± 14
Female gender	33%	33%
Unconjugated BR (μmol/l)	33.12 ± 9.86	9.22 ± 3.41**
Haem (μmol/l)	0.75 ± 0.14	0.76 ± 0.11
AST (U/l)	26.02 ± 8.31	25.43 ± 8.31
ALT (U/l)	23.56 ± 8.15	24.78 ± 9.07
GGT (U/l)	21.10 ± 15.04	23.78 ± 17.47
LDH (U/l)	160 ± 22	163 ± 29
CO-hemoglobin (%)	1.21 ± 0.35	1.32 ± 0.72
Haemoglobin (g/dl)	14.53 ± 1.30	14.27 ± 1.21
Haematocrit (%)	42 ± 3.37	41 ± 3.50
Iron (μmol/l)	30.26 ± 10.05	23.11 ± 9.48**
Transferrin (mg/dl)	266 ± 45	268 ± 40
Transferrin saturation (%)	45 ± 15.60	35 ± 14.14**
Ferritin (μg/l)	120 ± 111	120 ± 87
Haptoglobin (mg/dl)	78 ± 33	103 ± 51**
Hemopexin (mg/dl)	84 ± 10	87 ± 14*
Albumin (g/l)	47 ± 3	47 ± 3
Free BR (nmol/l)	2.20 ± 0.79	0.25 ± 0.23**
BMI (kg/m^2^)	22.81 ± 3.03	25.38 ± 4.91**
Lean body mass (%)	78 ± 6.51	75 ± 8.59*
Body fat (%)	22 ± 6.51	25 ± 8.59*
BP systolic (mmHg)	129 ± 13	133 ± 16
BP diastolic (mmHg)	67 ± 12	69 ± 12
Framingham risk score (%)^¥^	1.5 ± 2.9	2.3 ± 4.2*

Values are presented as mean ± standard deviation or percentage. The indices represent different levels of significance obtained using paired t-test or ^¥^Wilcoxon signed ranks test for non-parametric variables:

*P < 0.05; **P < 0.01 cases vs. controls.

The variable sample sizes presented are due to missing values in some of the parameters.
