# Supplementary material for: Longer telomeres in chronic, moderate, unconjugated hyperbilirubinaemia: insights from a human study on Gilbert’s Syndrome
Source: Sci Rep. 2016 Mar 1;6:22300. doi: 10.1038/srep22300 (PMC4772088; doi:10.1038/srep22300)
Supplement: Supplementary Information [file srep22300-s2.pdf]

Supplementary data for *"Longer telomeres in chronic, moderate, unconjugated hyperbilirubinaemia: insights from a human study on Gilbert's Syndrome"*

Tosevska A.[1]*, Moelzer C.[2], Wallner M.[2,3], Janosec M.[2], Schwarz U.[2], Kern C. [2,4], Marculescu R.[5], Doberer D.[6], Weckwerth W. [7,8], Wagner K. H.[1, 2]*

1 Research Platform Active Ageing, University of Vienna, Austria
2 Department of Nutritional Sciences, University of Vienna, Althanstrasse 14, 1090 Vienna, Austria
3 Institute of Dietetics and Nutrition, University of Applied Sciences, FH JOANNEUM, Alte Poststraße 149, 8020 Graz, Austria
4 Institute of Pharmacology, Medical University of Vienna, Waehringer Str. 13a, 1090 Vienna, Austria
5 Clinical Institute of Laboratory Medicine, Medical University of Vienna, Waehringer Guertel 18-20, 1090 Vienna, Austria,
6 Department of Clinical Pharmacology, Medical University of Vienna, Waehringer Guertel 18-20, 1090 Vienna, Austria
7 Department of Ecogenomics and Systems Biology, University of Vienna, Althanstrasse 14, 1090 Vienna, Austria
8 Vienna Metabolomics Center (VIME), University of Vienna, Austria

List of abbreviations:

BMI = Body mass index
BP = Blood pressure
CO-Hb = Carboxyhaemoglobin
CRP = C-reactive protein
FRAP = Ferric reducing ability of plasma
GSH/GSSG = Reduced/oxidized glutathione
HCY = Homocysteine
HOMA-IR = Homeostatic model assessment – insulin resistance
IL = Interleukin
MAP = Mean arterial pressure
MDA = Malondialdehyde
PLA2 = Phospholipase A2
SAA = Serum amyloid alpha
TL = Telomere Length
TNF = Tumor necrosis factor

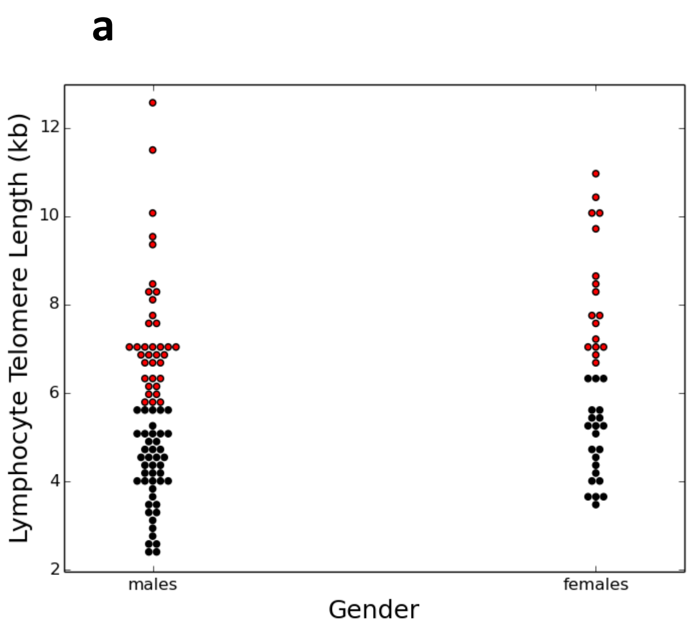

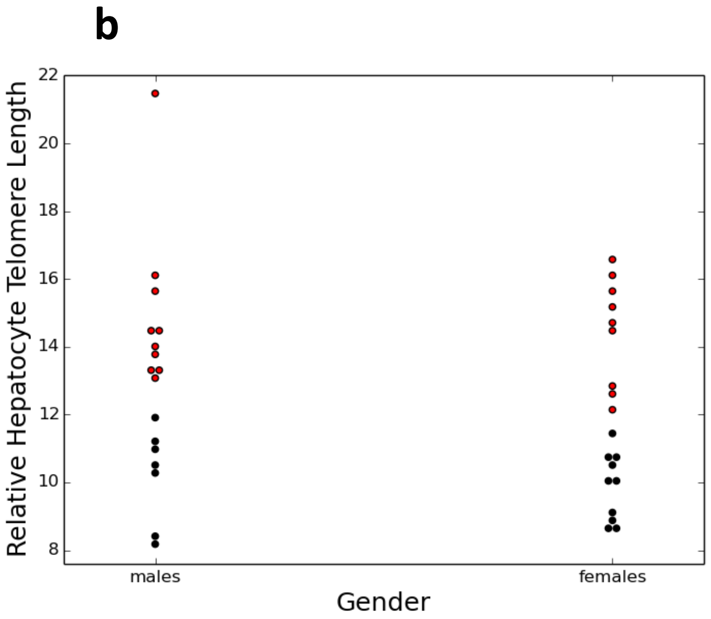

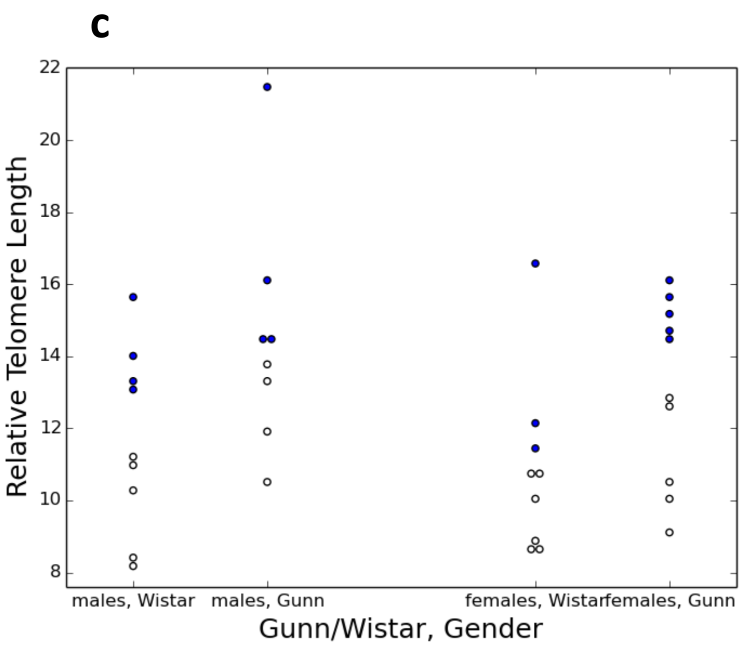

Supplementary figure S1. Gender differences in mean telomere length in a) Human lymphocytes ($P < 0.1$). b) Gunn and Wistar rats' hepatocytes (n.s.) c) Differences between males and females in Gunn and Wistar ruts separately.

Different colors represent values below and above the group mean.

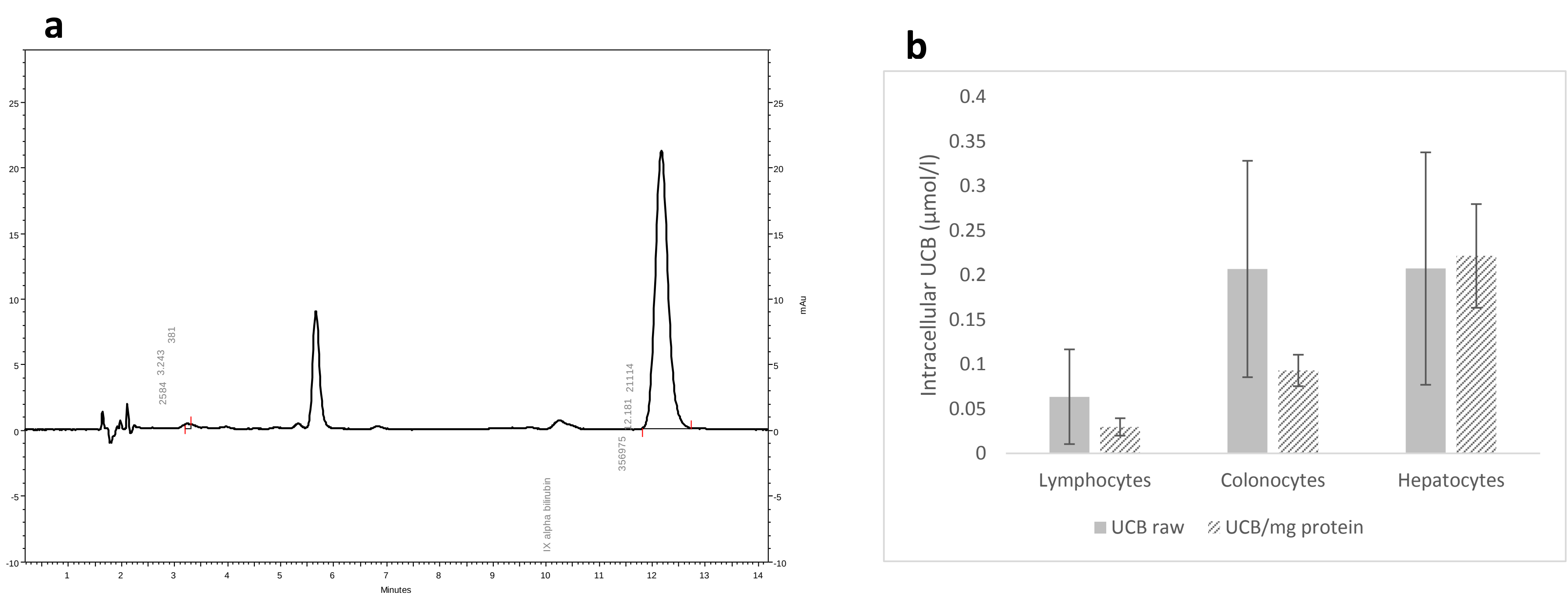

Supplementary figure S2. UCB measurements in human serum and rat lymphocytes, hepatocytes and colonocytes. a) A representative chromatogram of unconjugated bilirubin measurement in serum. Only IX-α Bilirubin isomer is detectable in serum samples and tissue samples derived from rats. No peak present in human lymphocytes. b) Intracellular content of UCB in Gunn rat lymphocytes, colonocytes and hepatocytes. Error bars represent standard deviation.

Supplementary Table S1. Gender differences in mean values between GS and non-GS individuals. Values are presented as mean ± standard deviation. The indices represent different levels of significance obtained using two-tailed independent samples T-test:

[a] P < 0.05, [aa] P < 0.01 when comparing females to males within the same condition (GS or control), *P < 0.05, **P < 0.01 when comparing GS to controls of the same gender, [b] P < 0.1 females compared to males within the same condition (GS or control), [#] P < 0.1 GS compared to controls of the same gender.

The variable sample sizes presented are due to missing values in some of the parameters.

	Control		*GS*	
	Males (n = 36-40)	Females (n = 19-20)	Males (n = 37-40)	Females (n = 18-20)
Age (years)	36 ± 14	41 ± 14	36 ± 14	41 ± 14
UCB (µmol/l)	9.65 ± 3.53	8.35 ± 3.05	35.21 ± 10.53**	28.94 ± 6.85[a]**
Lymph. TL (kb)	5.10 ± 2.00	6.19 ± 2.12[b]	6.20 ± 1.87*	6.52 ± 2.03
BMI (kg/m²)	25.74 ± 5.15	24.66 ± 4.42	23.34 ± 3.07*	21.75 ± 2.71[a]*
Body fat (%)	21.82 ± 7.37	30.76 ± 7.77[aa]	20.90 ± 6.05	24.42 ± 6.96 [b]*
CO-Hb (%)	1.42 ± 0.85	1.13 ± 0.30[b]	1.23 ± 0.30	1.18 ± 0.44
Heme (µmol/l)	0.77 ± 0.12	0.73 ± 0.09	0.74 ± 0.13	0.78 ± 0.18
Iron (µmol/l)	23.27 ± 8.98	22.79 ± 10.71	32.14 ± 10.73**	26.43 ± 7.32[a]
Albumin (g/l)	47.00 ± 3.18	45.75 ± 1.77[b]	48.06 ± 3.42	45.17 ± 2.15[aa]
Estradiol (pg/ml)	31.75 ± 9.91	117 ± 104.38[aa]	31.87 ± 11.71	70.20 ± 74.47[aa]
Testosterone (ng/ml)	5.60 ± 1.54	0.31 ± 0.14[aa]	6.12 ± 1.90	0.34 ± 0.13[aa]
HCY (µmol/l)	11.28 ± 3.87	8.77 ± 2.02[aa]	12.24 ± 5.20	9.28 ± 1.88[aa]
FRAP (mmol Fe^{2+}/l)	561 ± 91	518 ± 167	827 ± 170**	691 ± 169[aa]**
GSH/GSSG	1.15 ± 0.46	1.47 ± 0.32[aa]	1.11 ± 0.36	1.37 ± 0.42[a]
MDA (nmol/ml)	1.65 ± 0.55	1.95 ± 0.52[b]	1.75 ± 0.50	1.68 ± 0.47
IL6 (rfu)	2.58 ± 0.26	2.48 ± 0.31	2.35 ± 0.25**	2.40 ± 0.32
IL1β (rfu)	1.59 ± 0.28	1.41 ± 0.21[aa]	1.41 ± 0.23**	1.43 ± 0.33
TNF (rfu)	26.17 ± 5.24	25.13 ± 4.85	25.38 ± 5.92	26.37 ± 4.74

CRP (mg/dl)	0.17 ± 0.29	0.15 ± 0.18	0.10 ± 0.12	0.05 ± 0.05*
SAA (mg/dl)	4.62 ± 1.88	5.73 ± 2.78[b]	4.42 ± 1.34	4.31 ± 1.18*
Uric acid (mg/dl)	52.30 ± 18.36	38.85 ± 14.39[aa]	56.55 ± 14.52	40.15 ± 15.32[aa]
PLA2 (mg/dl)	32.60 ± 47.10	30.15 ± 42.34	43.65 ± 51.48	27.75 ± 34.20
BP systolic (mmHg)	136 ± 15	126 ± 15[a]	133 ± 11	123 ± 13[aa]
BP diastolic (mmHg)	71 ± 11	64.79 ± 11[b]	70.25 ± 10	60.94 ± 12[aa]
MAP (mmHg)	114 ± 12	105 ± 13[a]	111 ± 10	101 ± 11[aa]
HOMA_IR	1.84 ± 1.62	1.43 ± 0.90	1.11 ± 0.87*	1.00 ± 0.66[#]

Supplementary table S2. Gender differences in mean values between Gunn and Wistar rats. Values are presented as mean ± standard deviation. The indices represent different levels of significance obtained using two-tailed independent samples T-test:

[a] $P < 0.05$, [aa] $P < 0.01$comparing females to males within the same rodent model (Gunn/ Wistar), *$P < 0.05$, **$P < 0.01$ comparing Gunn to Wistar rats of the same gender, [b] $P < 0.1$ females to males within the same rodent model (Gunn/Wistar), [#] $P < 0.1$ comparing Gunn to Wistar rats of the same gender.

The variable sample sizes presented are due to missing values in some of the parameters.

	Wistar		*Gunn*	
	Males (n = 9)	Females (n = 9-11)	Males (n = 8-9)	Females (n = 10-11)
UCB (μmol/l)	0.58 ± 0.18	0.55 ± 0.20	97.88 ± 19.77**	112.38 ± 29.64**
Relative TL	11.66 ± 2.54	10.91 ± 2.47	14.46 ± 3.27[#]	13.12 ± 2.55[#]

Supplementary Table S3. Age differences in mean values in GS and non-GS individuals. Values are presented as mean ± standard deviation. The indices represent different levels of significance obtained using two-tailed independent samples T-test:

[a] P < 0.05, [aa] P < 0.01 when comparing individuals ≥ 35 years old to < 35 y.o. within the same condition (GS/control), *P < 0.05 comparing GS and control of the same age group, [b] P < 0.1 comparing individuals ≥ 35 years old to < 35 y.o. within the same condition (GS/control), [#] P < 0.1 comparing GS and control of the same age group.

The variable sample sizes presented are due to missing values in some of the parameters.

	Control		*GS*	
	< 35 y.o. (n = 30-33)	≥ 35 y.o. (n = 25-27)	< 35 y.o. (n = 30-33)	≥ 35 y.o. (n = 23-27)
Age (years)	27 ± 4	50 ± 11[aa]	27 ± 4	50 ± 11[aa]
UCB (µmol/l)	10.03 ± 3.63	8.22 ± 2.87[a]	33.57 ± 10.12**	32.57 ± 9.69**
Lymph. TL (kb)	5.83 ± 2.10	5.02 ± 2.01	6.57 ± 2.17	5.99 ± 1.52[#]
BMI (kg/m^2)	23.43 ± 3.74	27.77 ± 5.15[aa]	22.61 ± 3.01	23.06 ± 3.09**
Body fat (%)	21.30 ± 5.58	29.33 ± 9.61[aa]	22.23 ± 6.06	21.79 ± 7.12**
CO Hb (%)	1.15 ± 0.37	1.53 ± 0.97[a]	1.26 ± 0.43	1.14 ± 0.18*
Heme (µmol/l)	0.75 ± 0.11	0.77 ± 0.11	0.74 ± 0.13	0.77 ± 0.16
Iron (µmol/l)	25.47 ± 9.53	20.20 ± 8.75[a]	31.31 ± 10.45*	29.06 ± 9.63*
Albumin (g/l)	47.15 ± 3.07	45.88 ± 2.39[b]	47.91 ± 3.68	46.11 ± 2.59[a]
Estradiol (pg/ml)	62.00 ± 71.49	57.78 ± 74.29	38.58 ± 25.87[#]	52.85 ± 65.00
Testosterone (ng/ml)	4.51 ± 2.80	2.91 ± 2.62	4.86 ± 3.14	3.27 ± 3.00
HCY (µmol/l)	10.38 ± 3.95	10.56 ± 3.04	12.22 ± 5.68	10.11 ± 2.41[a]
FRAP (mmol Fe^{2+}/l)	568 ± 106	521 ± 139	806 ± 190**	748 ± 166**
GSH/GSSG	1.17 ± 0.45	1.37 ± 0.41[b]	1.17 ± 0.41	1.22 ± 0.40
MDA (nmol/ml)	1.68 ± 0.58	1.83 ± 0.52	1.77 ± 0.50	1.68 ± 0.46
IL-6 (rfu)	2.56 ± 0.30	2.54 ± 0.26	2.35 ± 0.26**	2.38 ± 0.29*
IL1-β (rfu)	1.53 ± 0.24	1.53 ± 0.31	1.43 ± 0.23	1.40 ± 0.30

TNF (rfu)	26.34	±	4.97	25.19	±	5.27	26.73	±	5.34	24.56	±	5.59
CRP (mg/dl)	0.10	±	0.10	0.25	±	0.35[a]	0.07	±	0.07	0.10	±	0.14*
SAA (mg/dl)	4.54	±	1.55	5.54	±	2.84[b]	4.18	±	0.86	4.64	±	1.65
Uric acid (mg/dl)	47.36	±	17.31	48.37	±	19.50	50.76	±	20.38	51.48	±	10.69
PLA2 (mg/dl)	33.61	±	44.39	29.56	±	46.97	38.12	±	49.48	38.63	±	44.11
BP systolic (mmHg)	131.10	±	16.71	134.44	±	14.58	128.97	±	12.73	131.19	±	13.56
BP diastolic (mmHg)	66.61	±	13.10	72.07	±	9.75[b]	66.06	±	9.55	68.96	±	14.38
MAP (mmHg)	109.17	±	14.00	113.24	±	11.32	107.58	±	10.57	110.03	±	12.19
HOMA_IR	1.22	±	0.77	2.32	±	1.79[aa]	1.00	±	0.49	1.17	±	1.05**

Supplementary table S4. Percentage of individuals carrying the GS-related (7/7) genotype among the 2 phenotypes: 6/6 - homozygotes with alleles containing 6 TA – repeats (short allele), 6/7 - heterozygotes with one short and one long allele, 7/7- homozygotes with two 7-TA long alleles.

		**GS Phenotype**		**Total %**
		no	yes	
**UGT1A1 Genotype**	6/6	43 %	2 %	26
	6/7	52 %	9 %	35
	7/7	5 %	89 %	54
**Total N**		58	57	115

Supplementary table S5. Descriptive values of UGT1A1 genotypes (6/6 wildtype homozygous, 6/7 heterozygous, 7/7 homozygous with reduced UGT1A1 activity). Values are expressed as mean ± standard deviation. *P < 0.05, **P < 0.01, #P < 0.1; Kruskal-Wallis ANOVA for nonparametric data.

	Genotype		
	6/6 (n = 25)	6/7 (n = 34)	7/7 (n = 52)
Age (years)	37 ± 14	36 ± 15	39 ± 14
Female gender (%)	31	31	35
UCB (µmol/l)**	9.27 ± 3.67	12.74 ± 9.07	31.93 ± 10.90
Lymphocyte telomere length (kb)*	5.22 ± 1.88	5.70 ± 2.27	6.33 ± 1.91
BMI (kg/m^2)#	25.32 ± 4.91	25.08 ± 4.87	23.12 ± 3.30
Iron (µmol/l)	23.94 ± 8.49	26.96 ± 12.44	27.84 ± 9.72

Supplementary table S6. Oligos used for qPCR-based telomere length measurement. Oligos a-e were designed according to (O'Callaghan and Fenech 2011).

Target/name	Species	Sequence (5'-3')	Amplicon size
**Telomere standard**[a]	Human/rodent	$(TTAGGG)_{14}$	84bp
**36B4 standard**[b]	Human	CAGCAAGTGGGAAGGTGTAATCCGTCTCCACAGACAAGGCCAGGAC TCGTTTGTACCCGTTGATGATAGAATGGG	75bp
**Telomere F primer**[c]	Human/rodent	CGGTTTGTTTGGGTTTGGGTTTGGGTTTGGG TTTGGGTT	>76 bp
**Telomere R primer**	Human/rodent	GGCTTGCCTTACCCTTACCCTTACCC TTACCCTTACCCT	
**36B4 F primer**[d]	Human	CAGCAAGTGGGAAGGTGTAATCC	75bp
**36B4 R primer**[e]	Human	CCCATTCTATCATCAACGGGTACAA	
**36B4 F primer**	Rodent	AGGTCGAAGCAAAGGAAGAGTCG	84bp
**36B4 R primer**	Rodent	CTGACTTGGTGTGAGGGGCTT	

References

O'Callaghan, N. J. and M. Fenech (2011). "A quantitative PCR method for measuring absolute telomere length." Biol Proced Online **13**: 3.
